# Post-vaccination T cell immunity to omicron

**DOI:** 10.3389/fimmu.2022.944713

**Published:** 2022-07-26

**Authors:** Henning Jacobsen, Viviana Cobos Jiménez, Ioannis Sitaras, Naor Bar-Zeev, Luka Čičin-Šain, Melissa M. Higdon, Maria Deloria-Knoll

**Affiliations:** ^1^ Department of Viral Immunology, Helmholtz Center for Infection Research, Braunschweig, Germany; ^2^ Military HIV Research Program, Walter Reed Army Institute of Research, Silver Spring, MD, United States; ^3^ Henry M. Jackson Foundation for the Advancement of Military Medicine, Bethesda, MD, United States; ^4^ W. Harry Feinstone Department of Molecular Microbiology and Immunology, Johns Hopkins Bloomberg School of Public Health, Baltimore, MD, United States; ^5^ International Vaccine Access Center, Department of International Health, Johns Hopkins Bloomberg School of Public Health, Baltimore, MD, United States; ^6^ Centre for Individualised Infection Medicine (CIIM), a joint venture of HZI and MHH, Hannover, Germany; ^7^ German Centre for Infection Research (DZIF), Hannover-Braunschweig site, Germany

**Keywords:** SARS-CoV-2, omicron, T cell, COVID-19, vaccine

## Abstract

In late 2021, the omicron variant of SARS Coronavirus 2 (SARS-CoV-2) emerged and replaced the previously dominant delta strain. Effectiveness of COVID-19 vaccines against omicron has been challenging to estimate in clinical studies or is not available for all vaccines or populations of interest. T cell function can be predictive of vaccine longevity and effectiveness against disease, likely in a more robust way than antibody neutralization. In this mini review, we summarize the evidence on T cell immunity against omicron including effects of boosters, homologous versus heterologous regimens, hybrid immunity, memory responses and vaccine product. Overall, T cell reactivity in post-vaccine specimens is largely preserved against omicron, indicating that vaccines utilizing the parental antigen continue to be protective against disease caused by the omicron variant.

## Introduction

By March 2022, nine different Coronavirus disease-19 (COVID-19) vaccines were authorized by WHO for clinical use. All current vaccine platforms utilize antigens derived from the parental SARS-CoV-2 virus that dominated initial pandemic waves (1). SARS-CoV-2 has demonstrated a potential for rapid mutations and numerous variants have evolved, starting approximately one year after its discovery (2). WHO classified variants of interest and concern (VoC) reflecting their biological and clinical relevance, including immune evasion (3). The first VoC, alpha, presented features of increased transmission, but vaccine effectiveness was sustained (4–6). Beta showed substantial immune escape *in vitro* and ability to spread in countries with high seroprevalence, but remained restricted to subregional outbreaks (7–9). When delta emerged in late 2020, it replaced other variants globally (10) due to its transmission advantage rather than an association with impaired vaccine effectiveness (4, 11–13). In November 2021, omicron raised strong concerns because of numerous amino acid mutations in the spike protein (14) and rapidly replaced delta in most countries and is now globally dominant (15). Omicron is significantly more transmissible compared to delta, and primary vaccine regimen and non-omicron infections appear to show considerably reduced effectiveness against omicron infection and symptomatic disease (16, 17).

Numerous studies based on virus neutralization assays, where the ability of immune sera to inhibit virus entry into cells is directly measured as correlate of vaccine effectiveness (18, 19), demonstrated a large drop in neutralizing titers against Omicron (10×->30×) compared to previous variants such as Beta (2×-10×) and delta (2×-4×). Furthermore, a high proportion of vaccinees had no detectable neutralizing antibodies against omicron (20–24). This effect was especially pronounced for inactivated and vector-based vaccines (20, 21, 24–26) and motivated global booster campaigns.

While neutralizing antibody titers strongly correlate with protection from infection, including symptomatic infection, the correlation is weaker for protection against severe disease and death, especially for immune-escaping variants (27–30). Robust T cell responses are associated with milder COVID-19 and they have been found to contribute to protective responses elicited by vaccination (31–35). CD8 T cell responses have been found to be an important contributor to protection against severe COVID-19, especially in the context of suboptimal antibody responses (31, 34, 36–38), while CD4 T cells are essential for protective antibody responses and an indispensable supporter of CD8 T cell maturation and proliferation (39).

Some SARS-CoV-2 specific T cells may persist long after antigenic clearance (40–42). Long-term T-cell immunity has been reported following infection and vaccination (29, 41, 43, 44). Notably, SARS-CoV-1 reactive T cells could be detected 17 years after infection (45).

In contrast to neutralizing antibody epitopes, epitopes for T cell recognition are distributed beyond the RBD domain of the spike protein and were found in multiple viral proteins. Hence, specific spike mutations in SARS-CoV-2 variants may not allow immune escape from T cell responses (46–49). Although early studies of SARS-CoV-2 VoCs have shown that the majority of CD4 and CD8 T cell responses are well preserved (50–53), the extent of T cell cross-reactivity to omicron remains unclear. Hence, understanding the generation and maintenance of robust and cross-reactive SARS-CoV-2-specific T cell responses is important to evaluate vaccine efficacy against SARS-CoV2 variants.

Studying cellular immunity in clinical specimens is technically and logistically more complex than measuring antibody responses because it requires intact cells. Cellular assays should be ideally performed directly after sample acquisition, or samples require rapid cryo-preservation, and Human Leukocyte Antigen (HLA) restriction modulates individual antigenic responses. Assays based on *in vitro* restimulation with antigens, such as ELISpots, Interferon Gamma Release Assays (IGRA) or Intracellular cytokine staining (ICS) and flow cytometry require viable cells, and complex processing over several hours or days (54). Typically, the stimulation is performed with peptide pools spanning the antigen of interest (usually the full-length spike protein, but the principle can be applied to any protein or part thereof). Cytokine release upon activation of T cells that recognize peptides present in the pool is measured, where Interferon gamma is the most common cytokine and is frequently used to identify antigen-specific responding cells. Both ICS and ELISpot allow the quantification of antigen specific T cells, where ICS allows assessment of a higher number cytokines in the same cell and it is more widely used. Tetramer staining of cells allows identification of antigen-specific cells in absence of *in vitro* restimulation but can only identify defined antigenic peptides in the context of presenting HLA molecules, and thus requires haplotyping. In this mini-review we will summarize the existing evidence on T cell specific immunity against the omicron VoC and its possible impact on vaccine effectiveness.

## CD4 and CD8 T cell reactivity against SARS-CoV-2 omicron

We reviewed studies comparing CD4 and/or CD8 T cell reactivity to omicron and parental-strain SARS-CoV-2 published before March 31^st^, 2022, to understand the effects of omicron on vaccine performance. Studies reviewed here followed similar methodology and reported data on omicron-specific CD4 and CD8 T cell response. A summary of all studies included is provided as **Supplementary Table 1**. The term ‘reactivity’ describes the potential of T cells to become activated upon stimulation with an antigen of interest. The ratio of T cells activated by omicron *vs* parental antigen are presented as ‘percent relative reactivity’ (can be >100%), as the main denominator of T cell cross-reactivity against omicron.

**Figure 1:**
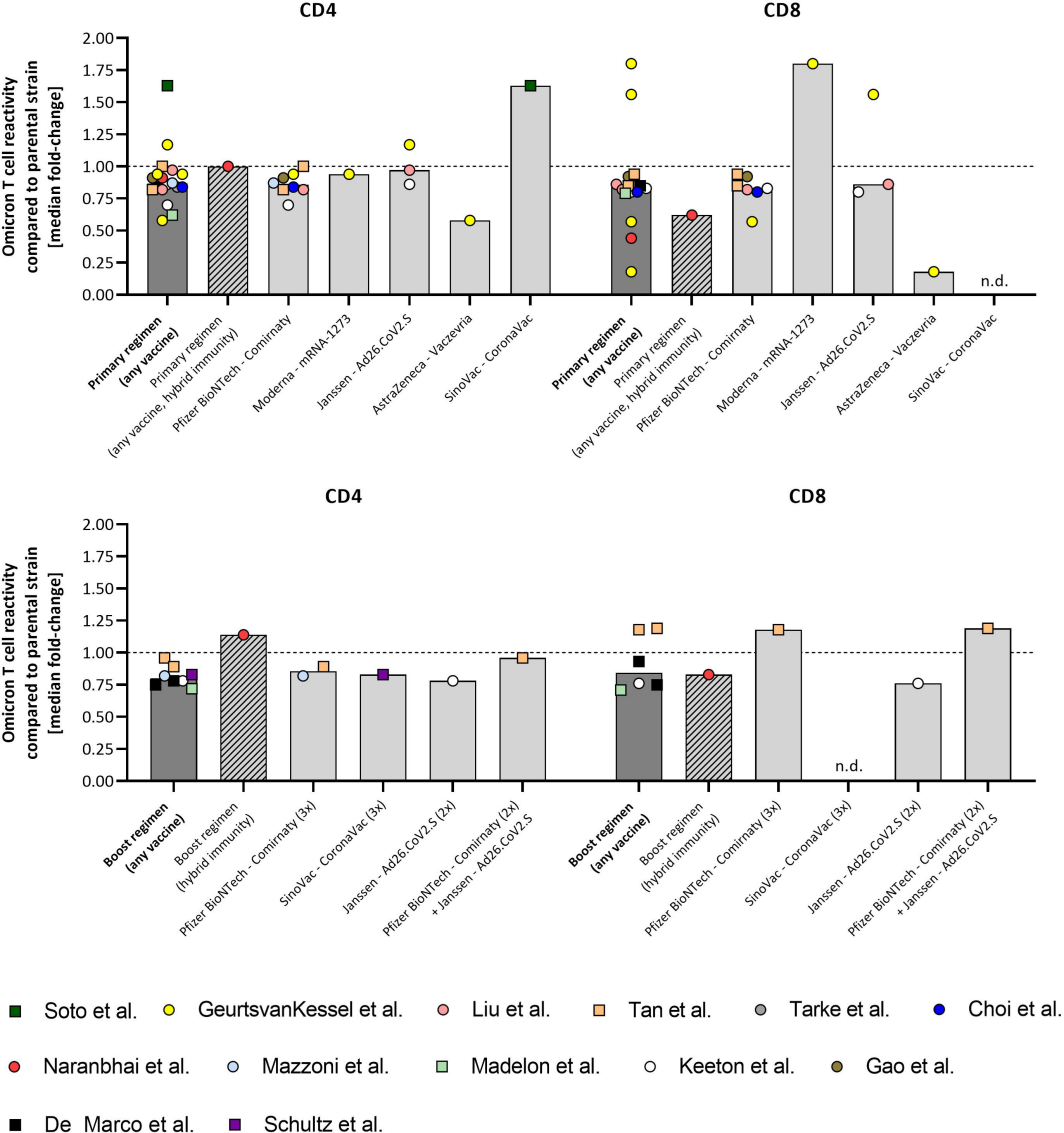
Overview of study results on omicron-specific T cell reactivity compared to the parental strain of SARS-CoV-2. Study results from 13 studies assessing omicron-specific T cell reactivity available by March 31^st^, 2022, are shown as fold-change compared to parental-specific T cell activation. Non peer-reviewed pre-prints are shown as squares. A) Data on primary vaccine regimen and B) data on booster regimen. Every data point represents a single study. Data points from the same study are depicted with the same color. Data are either presented within the manuscript or abstracted from high-resolution figures or raw data provided with the manuscript. “Primary regimen (any vaccine)” and “Boost regimen (any vaccine) show all data points from studies on primary or boost vaccine cohorts, respectively (dark grey bars), excluding hybrid immunity shown in a separate bar (shaded bars). Data are not stratified for sampling intervals and peak-immunity as well as waned immunity data sets are included. Bars indicate the median fold-change in T cell cross-reactivity to omicron, compared to the parental strain. N.d, no data.

Unless otherwise stated, primary vaccination indicates two doses of vaccine, except one dose for Janssen–Ad26.CoV2.S; booster indicates one additional dose. **Figure 1** displays all currently available study results that directly assessed CD4 and CD8 omicron-specific T cell responses compared to the parental antigen and that are discussed in this review. Non peer reviewed studies are included to this review and are differentiated from peer reviewed studies as squares in **Figure 1**.

### Primary series vaccination

Generally, T-cell recognition of Omicron antigens decreased modestly, if at all. Studies comparing omicron to parental-strain specific CD4 and/or CD8 T cell reactivity by ICS or AIM mostly showed largely preserved (>80%) cross-reactivity against omicron for both CD4 and CD8 T cells (55–59), although subject heterogeneity in responses was more pronounced for the CD8 subset (55–57, 60).

T cell cross-reactivity to omicron one month after primary immunization with Ad26.CoV2.S or Pfizer/BioNTech–BNT162b2 was maintained to a similar extent as after up to 6 months post immunization with two doses of mRNA vaccines (55, 56). A broader study that combined data from primary BNT162b2, Ad26.CoV2.S, Moderna–mRNA-1273 and AstraZeneca–Vaxzevria vaccination reported no major differences between Omicron and parental at early or late time points (60). Although Choi et al. did not report the time post primary immunization with BNT162b2, T cell cross-reactivity was similar (58). However, another study found approximately 20% of vaccinees displayed a two-fold or greater reduction in overall functional T cell reactivity (61).

### Booster vaccination

Boosted subjects generally had high functional T cell responses for both CD4 and CD8 and for both homologous and heterologous boost schedules, but a few subjects still showed considerably reduced (approximately 9%) functional T cell reactivity against omicron, and differences were found between studies in direction and magnitude of the booster effect relative to the primary series (61).

Three studies found reduced cross-reactivity to omicron in CD4 T cell responses after a boost compared to primary series. Within these studies, T cell cross-reactivity against omicron was found to be 70% to 87% for all vaccines and regimen tested but post-boost cross-reactivity was approximately 5 – 15% lower compared to post-prime measurements (55, 62, 63). Notably, this effect does not depict an overall reduction in T cell responses against omicron but rather a more pronounced boosting of overall responses against the parental spike compared to the omicron spike resulting in reduced ratios for cross-recognition.

Contrasting results from two other studies show that CD4 T cell reactivity assessed 6 months after primary vaccination was similar to that after both a boost (59) and GeurtsvanKessel et al. found in mRNA boosted subjects that CD4 and CD8 T cells were strongly cross-reactive to omicron and greatly exceeded T cell reactivity against the parental strain (60). Of note, in these studies T cell reactivity was measured ≥6 months after the primary vaccine regimen and compared to samples acquired one month post-boost. Since our understanding about waning (or maturing) T cell cross-reactivity to SARS-CoV-2 VoCs is limited, it is difficult to assess if these comparisons are ideal.

Only a single study assessed CD4 T cell immunity of booster immunization with an inactivated vaccine (Sinovac–CoronaVac), which found the relative response against omicron retained to 83%, but did not compare to primary vaccination (64). However, another study that assessed primary vaccination with CoronaVac in children found the CD4 T cell response against omicron was increased (163%) compared to stimulation with a parental peptide pool (65).

Studies of the booster effect on omicron-specific CD8 T cell responses also showed varying results. One study observed no differences (91% and 93% in boosted subjects *vs* 83% post primary series) (62), whereas another observed booster doses increased omicron-specific relative CD8 T cell responses from 85% after primary vaccination to 118% after homologous boost and from 94% to 119% after heterologous boost (59).

### Vaccine products

Comparing different vaccines between studies is not recommended given the heterogeneity between studies for the same vaccine as shown in the previous section above. But some evidence was found to suggest that cross-reactive T cell responses to omicron may differ by vaccine. In one study evaluating four different vaccine cohorts, sample sizes were small but CD4 T cell cross-reactivity to omicron was significantly reduced after primary Vaxzevria immunization (58%) compared to the Ad26.CoV2.S (117%), mRNA-1273 (94%), and BNT162b2 (94%) (60). CD8 T cell cross-reactivity was reduced for both Vaxzevria (18%) and BNT162b2 (57%) compared to Ad26.CoV2.S (156%) and mRNA-1273 (180%) (60). However, these data should be considered with caution because of small group sizes and technical limitations such as low sensitivity leading to weak responses.

### Convalescence

Two studies found that convalescent subjects showed similar T cell reactivity to vaccinated subjects (55, 61), and another study found lower T cell reactivity among convalescent subjects (57). Keeton et al. compared convalescent subjects to those primed or boosted and found similar relative omicron-specific responses for both CD4 (77% *vs* 70-86%) and CD8 (75% *vs* 76-83%) relative to parental peptides. Naranbhai et al. compared effector T cell reactivity using IFN-γ ELISpot assays between convalescent and vaccinated subjects, which was preserved in most individuals in both groups. Yet, both groups had approximately 20% of subjects with at least 50% reduction in overall functional T cell reactivity. Gao et al. found higher relative omicron-specific T cell reactivity in vaccinated subjects (91% and 92% for CD4 and CD8 T cells, respectively) than convalescent subjects (84% and 70%, respectively).

### Hybrid immunity

“Hybrid immunity” describes vaccinated subjects who have had one or more SARS-CoV-2 infections either before or after vaccination regardless of whether a primary vaccine regimen or booster was completed. These subjects generally showed similar results to vaccinated-only subjects when assessing spike and omicron specific responses. A study comparing vaccinated to hybrid immune subjects found effector T cell reactivity against omicron was preserved in most individuals in both cohorts, and both also had approximately 20% of subjects with a pronounced reduction in overall functional T cell reactivity against omicron (61). One pre-print study found comparable reductions in T cell reactivity against omicron between hybrid immune subjects and homologous primary vaccinated subjects; both showed a small but significant reduction in CD4 (84%) and CD8 (83%) T cell reactivity against omicron (62). The timing of COVID-19 infection relative to vaccination was also explored (data from single-dose to heterologous booster were combined); subjects immunized after infection retained 82% CD4 T cell reactivity to omicron as compared to 85% among subjects infected after vaccination, and for CD8 T cell reactivity was 89% when infected before versus 94% after vaccination (62).

### T cell memory

One study found CD4 memory T cell responses against omicron were retained against omicron in three different immune cohorts (primary series vaccinees: 91%, hybrid immune subjects: 100%, and boosted hybrid immune subjects: 114%), but CD8 T cell memory responses were markedly reduced in all (44%, 62% and 83%, respectively) (61). In another study, T cell reactivity eight months post immunization was largely conserved against omicron compared to parental strain for both BNT162b2 (CD4: 91%; CD8: 86%) and Ad26.CoV2.S (CD4: 84%; CD8: 82%) (66). Additionally, CD4 and CD8 populations after Ad26.CoV2.S vaccination were fully cross-reactive to omicron-specific peptide pools, which was seen in both long-term memory cells (central memory) and recently activated effector cells (66).

### The immunocompromised host

Few data are available for immunocompromised subjects. In anti-CD20-treated multiple sclerosis patients who received two or three doses of mRNA vaccines, omicron-specific cross-reactivity was lower than in healthy cohorts (67). However, the cross-reactivity of CD4 and CD8 T cells was not largely diminished after primary vaccination (CD4 T cell responses were 62% and CD8 were 79%). While the booster dose drastically enhanced overall T cell responses against both the parental strain and omicron, cross-reactivity post-booster dose (CD4: 72%; CD8: 71%) was similar to post-primary cross-reactivity.

## Discussion

The development of novel vaccines requires robust predictors of vaccine induced immunity and effectiveness that comprehensively addresses both humoral (B cell mediated) and cellular (T cell mediated) immunity. Robust protective responses are therefore a result of T cell and B cell responses that complement each other. Antibody responses are crucial to mediate neutralization and antibody-dependent cellular functions against viruses and most important for clearance of extracellular pathogens. While strong humoral responses are indicators of protection from infection and transmission, T cells mediate or “help” such antibody responses (CD4 T helper cells) as well as protect from severe disease by clearing all infected cells (CD8 cytotoxic T cells) (68, 69).

Antibody titers decline rapidly, and novel variants escape recognition, but vaccine effectiveness against severe disease and death by SARS-CoV-2 is maintained. Current SARS-CoV-2 vaccines have shown good and stable cellular responses for CD4 and CD8 T cells (66). Cellular immunity plays an important role in vaccine-mediated protection against disease and may inform the development and the optimization of vaccines against SARS-CoV-2 variants. It is therefore important to understand how T cell responses can provide protection against development of disease when new SARS-CoV-2 variants arise.

Our review of initial studies found that both CD4 and CD8 T cell cross-reactivity against omicron is largely preserved, to about 80% when compared to reactivity against the vaccine seed strain, contrasting sharply with the pronounced losses of neutralizing activity, but consistent with previous studies on earlier SARS-CoV-2 VoCs (49, 51, 53). This possibly reflects a comparably low evolutionary advantage for the virus to escape T cell recognition. One possibility is that T cells may play only a minor role in SARS-CoV-2 control, thus reducing the selection pressure for mutations of T cell epitopes. A more plausible explanation is that T cell epitopes are recognized in the context of the immensely heterogeneous HLA molecules. Hence, mutations of T cell epitopes provide an advantage to the virus only among the fraction of the people that share the same HLA. T cell responses against Influenza A virus mostly confer protection against severe disease. Escape from CD8 T cell responses go along with a high fitness cost for the virus, however, single mutations may provide only a small selective advantage, in particular in populations with multiple HLA alleles (70). Therefore, T cell escape mutations take very long to get established in a virus population and thus it would take much longer for a virus to evolve vaccine induced T cell immunity.

T cell immune escape has been well documented in chronic virus infections, such as HIV or HCV, where T cell escape provides a benefit to the virus in a single host, but it is less likely in the scenario of acute v infections that require continuous transmission to new hosts.

This review focused on cross-reactivity to understand the role of vaccination in eliciting protective immune responses against omicron. Currently, there are insufficient studies to compare the cross-reactivity of different vaccines. We did not review vaccination effects on overall cellular responses, and it should not be assumed that all vaccine regimens would perform equally. Indeed, studies found that booster doses seem to be strong inducers of T cell responses. Most data were for primary vaccination with BNT162b2 and present a homogenous picture of well-conserved CD4 and CD8 T cell reactivity against omicron. Although limited data were available for Ad26.CoV2.S for which results were similar. For most other vaccine regimens and immunological scenarios such as hybrid immunity, only one or two studies were available at this point.

Nevertheless, broad cross-reactivity of T cell immunity against omicron is indicative of preserved vaccine effectiveness against severe disease (19, 29, 34, 35), underlining the importance of cellular immunity on vaccine performance. Indeed, first reports show that vaccine effectiveness seems largely preserved against hospitalization due to omicron (4, 71, 72).

Importantly, several studies found that while CD4 T cell cross-reactivity against omicron is usually homogenously conserved among subjects, CD8 T cell cross-reactivity appears to be significantly reduced in subsets of individuals within the same cohort. A possible explanation for reduced T cell reactivity in some subjects was provided by a detailed analysis that mapped binding affinities of all parental and omicron spike peptides (8 – 11mers) to 150 HLA-A, -B and -C alleles; approximately 7% of omicron epitopes with sequences different from parental were predicted to result in reduced binding to one or more HLA alleles, and subsequently decreased epitope presentation (61). This decrease in epitope presentation may provide a possible explanation for reduced T cell reactivity in the population harboring such HLA alleles. However, in another study, no specific HLA alleles significantly correlating to omicron recognition could be identified by applying similar tools to detect possible HLA associations in omicron non-responders (56). Nevertheless, further decline of the cross-reactivity of T cell responses to other VoCs could translate into lower protection from severe disease over time. It is important to continue to monitor the duration and cross-reactivity of T cell responses induced by different vaccine platforms, to determine if a T-cell inducing vaccination/booster would be beneficial to make both cellular and humoral responses long-lasting and eliminate the need for regular vaccination (68). In particular, combination of different vaccination strategies may elicit strong tissue-resident T cell responses in the mucosa, as well as mucosal antibodies, that could confer long-lasting protection from immune-evasive VoCs (73), also highlighting the importance of mucosal responses to protect from respiratory viral pathogens (74).

In summary, T cell immunity to omicron is highly conserved in all vaccinees, and cross-reactivity is comparable among all cohorts tested. Yet, while cross-variant T-cell reactivity is mostly unaffected, overall strength of the response may vary and can be significantly enhanced by hybrid immunity or booster doses. T-cell immunity to SARS-CoV-2 remains only partially understood but is a dynamic and rapidly evolving research field. Novel insights in cellular immunity may provide crucial insight to improve vaccine performance against the omicron VoC and its rapidly evolving subvariants.

## Author contributions

HJ performed the initial literature review, collected and summarized data and wrote the manuscript. VJ, IS, NB-Z, LC-S, MH and MD-K reviewed parts of the literature and revised the manuscript. MH and MD-K acquired funding. All authors contributed to the article and approved the submitted version.

## Funding

This project was funded by the Coalition for Epidemic Preparedness Innovations.

## Conflict of interest

The authors declare that the research was conducted in the absence of any commercial or financial relationships that could be construed as a potential conflict of interest.

## Publisher’s note

All claims expressed in this article are solely those of the authors and do not necessarily represent those of their affiliated organizations, or those of the publisher, the editors and the reviewers. Any product that may be evaluated in this article, or claim that may be made by its manufacturer, is not guaranteed or endorsed by the publisher.

## References

[1] WHO. Coronavirus disease (COVID-19): Vaccines . Available at: https://www.who.int/news-room/questions-and-answers/item/coronavirus-disease-(covid-19)-vaccines.

[2] Walker AS Gethings O Pritchard E Jones J House T Bell I. Tracking the emergence of SARS-CoV-2 alpha variant in the united kingdom. N Engl J Med (2021) 385:2582–5. doi: 10.1056/NEJMc2103227 PMC8693687 34879193

[3] WHO. Tracking SARS-CoV-2 variants . Available at: https://www.who.int/health-topics/nipah-virus-infection/tracking-SARS-CoV-2-variants.

[4] Higdon MM. A Systematic Review of Coronavirus Disease 2019 Vaccine Efficacy and Effectiveness Against Severe Acute Respiratory Syndrome Coronavirus 2 Infection and Disease. Open Forum Infectious Diseases (2022) 9(6):ofac138. doi: 10.1093/ofid/ofac138 35611346 PMC9047227

[5] Donnelly MAP Chuey MR Soto R Schwartz NG Chu VT Konkle SL. Household transmission of SARS-CoV-2 alpha variant – United States, 2021. Clin Infect Dis (2022), ciac125. doi: 10.1093/cid/ciac125 35147176 PMC9047162

[6] Ulrich L Halwe NJ Taddeo A Ebert N Schön J Devisme C. Enhanced fitness of SARS-CoV-2 variant of concern alpha but not beta. Nature (2022) 602:307–13. doi: 10.1038/s41586-021-04342-0 PMC8828469 34937050

[7] Jacobsen H Sitaras I Higdon M Knoll MD Zeev NB Jurgensmeyer M. Results of studies evaluating the impact of SARSCoV-2 variants of concern on COVID-19 vaccines: An ongoing systematic review. View-hub (2021).

[8] Callaway E. Fast-spreading COVID variant can elude immune responses. Nature (2021) 589:500–1. doi: 10.1038/d41586-021-00121-z 33479534

[9] Singer SR Angulo FJ Swerdlow DL McLaughlin JM Hazan I Ginish N. Effectiveness of BNT162b2 mRNA COVID-19 vaccine against SARS-CoV-2 variant beta (B.1.351) among persons identified through contact tracing in Israel: A prospective cohort study. eClinicalMedicine (2021) 42. doi: 10.1016/j.eclinm.2021.101190 PMC8628463 34870134

[10] WHO. COVID-19 weekly epidemiological update, edition 58. (2021) (Geneva: World Health Organization).

[11] Hart WS Miller E Andrews NJ Waight P Maini PK Funk S. Generation time of the alpha and delta SARS-CoV-2 variants: An epidemiological analysis. Lancet Infect Dis (2022). doi: 10.1101/2021.10.21.21265216 PMC8843191 35176230

[12] Kuzmina A Wattad S Khalaila Y Ottolenghi A Rosental B Engel S. SARS CoV-2 delta variant exhibits enhanced infectivity and a minor decrease in neutralization sensitivity to convalescent or post-vaccination sera. iScience (2021) 24:103467. doi: 10.1016/j.isci.2021.103467 34805783 PMC8591850

[13] Davis C Logan N Tyson G Orton R Harvey WT Perkins JS. Reduced neutralisation of the delta (B.1.617.2) SARS-CoV-2 variant of concern following vaccination. PLoS Pathog (2021) 17:e1010022. doi: 10.1371/journal.ppat.1010022 34855916 PMC8639073

[14] WHO. Classification of omicron (B.1.1.529): SARS-CoV-2 variant of concern (2021). Available at: https://www.who.int/news/item/26-11-2021-classification-of-omicron-(b.1.1.529)-sars-cov-2-variant-of-concern.

[15] WHO. Weekly epidemiological update on COVID-19. (2022) (Geneva: World Health Organization).

[16] Fan Y Li X Zhang L Wan S Zhang L Zhou F. SARS-CoV-2 omicron variant: Recent progress and future perspectives. Sig Transduct Target Ther (2022) 7:1–11. doi: 10.1038/s41392-022-00997-x PMC9047469 35484110

[17] WHO. Weekly epidemiological update on COVID-19. (2022) (Geneva: World Health Organization).

[18] Krammer F. A correlate of protection for SARS-CoV-2 vaccines is urgently needed. Nat Med (2021) 27:1147–8. doi: 10.1038/s41591-021-01432-4 34239135

[19] Feng S Phillips DJ White T Sayal H Aley PK Bibi S. Correlates of protection against symptomatic and asymptomatic SARS-CoV-2 infection. Nat Med (2021) 27:2032–40. doi: 10.1038/s41591-021-01540-1 PMC8604724 34588689

[20] Cameroni E Bowen JE Rosen LE Saliba C Zepeda SK Culap K. Broadly neutralizing antibodies overcome SARS-CoV-2 omicron antigenic shift. Nature (2022) 602:664–70. doi: 10.1038/s41586-021-04386-2 PMC9531318 35016195

[21] Jacobsen H Strengert M Maaß H Ynga Durand MA Kessel B Harries M. Diminished neutralization responses towards SARS-CoV-2 omicron VoC after mRNA or vector-based COVID-19 vaccinations. (2022). doi: 10.1101/2021.12.21.21267898 PMC9673895 36400804

[22] Edara V-V Manning KE Ellis M Lai L Moore KM Foster SL. mRNA-1273 and BNT162b2 mRNA vaccines have reduced neutralizing activity against the SARS-CoV-2 omicron variant. Cell Rep Med (2022) 3:100529. doi: 10.1016/j.xcrm.2022.100529 35233550 PMC8784612

[23] van Gils MJ Ayesha Lavell AH van der Straten K Appelman B Bontjer I Poniman M. Antibody responses against SARS-CoV-2 variants induced by four different SARS-CoV-2 vaccines. (2022). doi: 10.1101/2021.09.27.21264163 PMC9113667 35580156

[24] Cheng SMS Mok CKP Leung YWY NG SS Chan KCK Ko FW. Neutralizing antibodies against the SARS-CoV-2 omicron variant BA.1 following homologous and heterologous CoronaVac or BNT162b2 vaccination. Nat Med (2022) 28:486–9. doi: 10.1038/s41591-022-01704-7 PMC8940714 35051989

[25] Syed AM Ciling A Khalid MM Sreekumar B Chen PY Kumar GR. Omicron mutations enhance infectivity and reduce antibody neutralization of SARS-CoV-2 virus-like particles. (2022). doi: 10.1101/2021.12.20.21268048 PMC9351483 35858386

[26] Wang K Jia Z Bao L Cai L Chi H Hu Y. Memory b cell repertoire from triple vaccinees against diverse SARS-CoV-2 variants. Nature (2022) 603:919–25. doi: 10.1038/s41586-022-04466-x PMC8967717 35090164

[27] Moss P. The T cell immune response against SARS-CoV-2. Nat Immunol (2022) 23:186–93. doi: 10.1038/s41590-021-01122-w 35105982

[28] Khoury DS Cromer D Reynaldi A Schlub TE Wheatley AK Juno JA. Neutralizing antibody levels are highly predictive of immune protection from symptomatic SARS-CoV-2 infection. Nat Med (2021) 27:1205–11. doi: 10.1038/s41591-021-01377-8 34002089

[29] Sette A Crotty S. Adaptive immunity to SARS-CoV-2 and COVID-19. Cell (2021) 184:861–80. doi: 10.1016/j.cell.2021.01.007 PMC7803150 33497610

[30] Niessl J Sekine T Buggert M. T Cell immunity to SARS-CoV-2. Semin Immunol (2021) 55:101505. doi: 10.1016/j.smim.2021.101505 34711489 PMC8529278

[31] Tan AT Linster M Tan CW Le Bert N Cia WN Kunasegaran K. Early induction of functional SARS-CoV-2-Specific T cells associates with rapid viral clearance and mild disease in COVID-19 patients. Cell Rep (2021) 34:108728. doi: 10.1016/j.celrep.2021.108728 33516277 PMC7826084

[32] Gagne M Corbett KS Flynn JF Foulds KE Wagner DA Andrew SF. Protection from SARS-CoV-2 delta one year after mRNA-1273 vaccination in nonhuman primates is coincident with an anamnestic antibody response in the lower airway. (2021). doi: 10.1101/2021.10.23.465542 PMC8639396 34921774

[33] Israelow B Mao T Klein J Song E Menasche B Omer SB. Adaptive immune determinants of viral clearance and protection in mouse models of SARS-CoV-2. Sci Immunol (2021) 6:eabl4509. doi: 10.1126/sciimmunol.abl4509 34623900 PMC9047536

[34] McMahan K Yu J Mercado NB Loos C Tostanoski LH Chandrashekar A. Correlates of protection against SARS-CoV-2 in rhesus macaques. Nature (2021) 590:630–4. doi: 10.1038/s41586-020-03041-6 PMC7906955 33276369

[35] Moderbacher CR Ramirez SI Dan JM Grifoni A Hastie KM Weiskopf D. Antigen-specific adaptive immunity to SARS-CoV-2 in acute COVID-19 and associations with age and disease severity. Cell (2020) 183:996–1012.e19. doi: 10.1016/j.cell.2020.09.038 33010815 PMC7494270

[36] Liao M Liu Y Yuan J Wen Y Xu G Zhao J. Single-cell landscape of bronchoalveolar immune cells in patients with COVID-19. Nat Med (2020) 26:842–4. doi: 10.1038/s41591-020-0901-9 32398875

[37] Peng Y Mentzer AJ Liu G Yao X Yin Z Dong D. Broad and strong memory CD4+ and CD8+ T cells induced by SARS-CoV-2 in UK convalescent individuals following COVID-19. Nat Immunol (2020) 21:1336–45. doi: 10.1038/s41590-020-0782-6 PMC7611020 32887977

[38] Sekine T Perez-Pozzi A Rivera-Ballesteros O Stralin K Gorin J-P Olsson A. Robust T cell immunity in convalescent individuals with asymptomatic or mild COVID-19. Cell (2020) 183:158–68.e14. doi: 10.1016/j.cell.2020.08.017 32979941 PMC7427556

[39] Painter MM Mathew D Goel RR Apostolidis SA Pattekar A Kuthuru O. Rapid induction of antigen-specific CD4+ T cells is associated with coordinated humoral and cellular immunity to SARS-CoV-2 mRNA vaccination. Immunity (2021) 54:2133–42.e3. doi: 10.1016/j.immuni.2021.08.001 34453880 PMC8361141

[40] Cohen KW Linderman SL Moodie Z Czartoski J Lai L Mantus G. Longitudinal analysis shows durable and broad immune memory after SARS-CoV-2 infection with persisting antibody responses and memory b and T cells. Cell Rep Med (2021) 2:100354. doi: 10.1016/j.xcrm.2021.100354 34250512 PMC8253687

[41] Dan JM Mateus J Kato Y Hastie KM Yu ED Faliti CE. Immunological memory to SARS-CoV-2 assessed for up to 8 months after infection. Science (2021) 371:eabf4063. doi: 10.1126/science.abf4063 33408181 PMC7919858

[42] Adamo S Michler J Zurbuchen Y Cervia C Taeschler P Raeber ME. Signature of long-lived memory CD8+ T cells in acute SARS-CoV-2 infection. Nature (2022) 602:148–55. doi: 10.1038/s41586-021-04280-x PMC8810382 34875673

[43] Goel RR Painter MM Apostolidis SA Mathew D Meng W Rosenfeld AM. mRNA vaccines induce durable immune memory to SARS-CoV-2 and variants of concern. Science (2021) 374:abm0829. doi: 10.1126/science.abm0829 34648302 PMC9284784

[44] Barouch DH Stephenson KE Sadoff J Yu J Chang A Gebre M. Durable humoral and cellular immune responses 8 months after Ad26.COV2.S vaccination. New Engl J Med (2021) 385:951–3. doi: 10.1056/NEJMc2108829 PMC8314733 34260834

[45] Le Bert N Tan AT Kunasegaran K Tham CYL Hafezi M Chia A. SARS-CoV-2-Specific T cell immunity in cases of COVID-19 and SARS, and uninfected controls. Nature (2020) 584:457–62. doi: 10.1038/s41586-020-2550-z 32668444

[46] Redd AD Nardin A Kared H Block EM Abel B Pekosz A. Minimal cross-over between mutations associated with omicron variant of SARS-CoV-2 and CD8+ T cell epitopes identified in COVID-19 convalescent individuals. bioRxiv (2021). doi: 10.1101/2021.12.06.471446 PMC8941890 35229637

[47] Ahmed SF Quadeer AA McKay MR. SARS-CoV-2 T cell responses are expected to remain robust against omicron. (2021). doi: 10.1101/2021.12.12.472315 PMC8781795 35062283

[48] Grifoni A Weiskopf D Ramirez SI Mateus J Dan JM Moderbacher CR. Targets of T cell responses to SARS-CoV-2 coronavirus in humans with COVID-19 disease and unexposed individuals. Cell (2020) 181:1489–501.e15. doi: 10.1016/j.cell.2020.05.015 32473127 PMC7237901

[49] Alter G Yu J Liu J Chandrashekar A Borducchi EN Tostanoski EN. Immunogenicity of Ad26.COV2.S vaccine against SARS-CoV-2 variants in humans. Nature (2021) 596:268–72. doi: 10.1038/s41586-021-03681-2 PMC8357629 34107529

[50] Collier AY Brown CM McMahan K Yu J Liu J Jacob-Dolan C. Immune responses in fully vaccinated individuals following breakthrough infection with the SARS-CoV-2 delta variant in provincetown, Massachusetts. (2021). doi: 10.1101/2021.10.18.21265113 PMC8995036 35258323

[51] Geers D Shamier MC Bogers S Hartog GD Gommers L Nieuwkoop NN. SARS-CoV-2 variants of concern partially escape humoral but not T cell responses in COVID-19 convalescent donors and vaccine recipients. Sci Immunol (2021) 6:eabj1750. doi: 10.1126/sciimmunol.abj1750 34035118 PMC9268159

[52] Keeton R Richardson SI Moyo-Gwete T Hermanus T Tincho MB Benede M. Prior infection with SARS-CoV-2 boosts and broadens Ad26.COV2.S immunogenicity in a variant-dependent manner. Cell Host Microbe (2021) 29:1611–19.e5. doi: 10.1016/j.chom.2021.10.003 34688376 PMC8511649

[53] Tarke A Sidney J Method N Yu ED Zhang Y Dan JM. Impact of SARS-CoV-2 variants on the total CD4+ and CD8+ T cell reactivity in infected or vaccinated individuals. Cell Rep Med (2021) 2:100355. doi: 10.1016/j.xcrm.2021.100355 34230917 PMC8249675

[54] Cossarizza A Chang H-D Radbruch A Abrignani S Addo R Akdis M. Guidelines for the use of flow cytometry and cell sorting in immunological studies (Third edition). Eur J Immunol (2021) 51:2708–3145. doi: 10.1002/eji.202170126 34910301 PMC11115438

[55] Keeton R Tincho MB Ngomti A Baguma R Benede N Suzuki N. T Cell responses to SARS-CoV-2 spike cross-recognize omicron. Nature (2022) 603:488–92. doi: 10.1038/s41586-022-04460-3 PMC8930768 35102311

[56] Tarke A Coelho CH Zhang Z Dan JM Yu ED Method N. SARS-CoV-2 vaccination induces immunological T cell memory able to cross-recognize variants from alpha to omicron. Cell (2022) 185:847–59.e11. doi: 10.1016/j.cell.2022.01.015 35139340 PMC8784649

[57] Gao Y Cai C Grifoni A Müller TR Niessl J Olofsson A. Ancestral SARS-CoV-2-Specific T cells cross-recognize the omicron variant. Nat Med (2022) 28:472–6. doi: 10.1038/s41591-022-01700-x PMC8938268 35042228

[58] Choi SJ Kim D-U Noh JY Kim S Park S-H Jeong HW. T Cell epitopes in SARS-CoV-2 proteins are substantially conserved in the omicron variant. Cell Mol Immunol (2022) 19:447–8. doi: 10.1038/s41423-022-00838-5 PMC8764507 35043006

[59] Tan CS Collier AY Liu J Yu J Chandrashekar A McMahan K. Homologous and heterologous vaccine boost strategies for humoral and cellular immunologic coverage of the SARS-CoV-2 omicron variant. (2021). doi: 10.1101/2021.12.02.21267198

[60] GeurtsvanKessel CH Geers D Schmitz SK Myktyn AZ Lamers MM Bogers S. Divergent SARS-CoV-2 omicron–reactive T and b cell responses in COVID-19 vaccine recipients. Sci Immunol 7:eabo2202. doi: 10.1126/sciimmunol.abo2202 PMC8939771 35113647

[61] Naranbhai V Nathan A Kaseke C Berrios C Khatri A Choi S. T Cell reactivity to the SARS-CoV-2 omicron variant is preserved in most but not all individuals. Cell (2022) 185:1041–51.e6. doi: 10.1016/j.cell.2022.01.029 35202566 PMC8810349

[62] De Marco L D'Orso S Pirronello M Verdiani A Termine A Fabrizio C. Preserved T cell reactivity to the SARS-CoV-2 omicron variant indicates continued protection in vaccinated individuals. (2021). doi: 10.1101/2021.12.30.474453

[63] Mazzoni A Vanni A Spinicci M Capone M Lamaccia G Salvati L. SARS-CoV-2 spike-specific CD4+ T cell response is conserved against variants of concern, including omicron. Front Immunol (2022) 13:801431. doi: 10.3389/fimmu.2022.801431 35154116 PMC8826050

[64] Schultz BM Melo-Gonzalez F Duarte LF Galvez NMS Pacheco GA Soto JA. A booster dose of an inactivated SARS-CoV-2 vaccine increases neutralizing antibodies and T cells that recognize delta and omicron variants of concern. (2022). doi: 10.1101/2021.11.16.21266350 PMC9426482 35946814

[65] Soto JA Melo-Gonalez F Gutierrez-Vera C Schultz BM Berrios-Rojas RV Rivera-Perez D. An inactivated SARS-CoV-2 vaccine is safe and induces humoral and cellular immunity against virus variants in healthy children and adolescents in Chile. (2022). doi: 10.1101/2022.02.15.22270973

[66] Liu J Chandrashekar A Sellers D Barrett J Jacob-Dolan C Lifton M. Vaccines elicit highly conserved cellular immunity to SARS-CoV-2 omicron. Nature (2022) 603:493–6. doi: 10.1038/s41586-022-04465-y PMC8930761 35102312

[67] Madelon N Heikkila N Royo IS Fontannaz P Breville G Lauper K. Omicron-specific cytotoxic T-cell responses are boosted following a third dose of mRNA COVID-19 vaccine in anti-CD20-Treated multiple sclerosis patients. (2021). doi: 10.1101/2021.12.20.21268128 PMC9002341 35212717

[68] Kingstad-Bakke B Lee W Chandrasekar S Gasper DJ Salas-Quinchucua C Cleven T. Vaccine-induced systemic and mucosal T cell immunity to SARS-CoV-2 viral variants. Proc Natl Acad Sci (2022) 119:e2118312119. doi: 10.1073/pnas.2118312119 35561224 PMC9171754

[69] Gilbert SC. T-Cell-Inducing vaccines – what’s the future. Immunology (2012) 135:19–26. doi: 10.1111/j.1365-2567.2011.03517.x 22044118 PMC3246649

[70] Li Z-RT Zarnitsyna VI Lowen AC Weissman D Koelle K Kohlmeier JE. Why are CD8 T cell epitopes of human influenza a virus conserved? J Virol (2019) 93. doi: 10.1128/JVI.01534-18 PMC6401462 30626684

[71] Collie S Champion J Moultrie H Bekker L-G Gray G. Effectiveness of BNT162b2 vaccine against omicron variant in south Africa. New Engl J Med (2022) 386:494–6. doi: 10.1056/NEJMc2119270 PMC8757569 34965358

[72] Gray GW Collie S Garrett N Coga A Champion J Zylstra M. Vaccine effectiveness against hospital admission in south African health care workers who received a homologous booster of Ad26.COV2 during an omicron COVID19 wave: Preliminary results of the sisonke 2 study. (2021). doi: 10.1101/2021.12.28.21268436

[73] Lapuente D Fuchs J Willar J Antao AV Eberlein V Uhlig N. Protective mucosal immunity against SARS-CoV-2 after heterologous systemic prime-mucosal boost immunization. Nat Commun (2021) 12:6871. doi: 10.1038/s41467-021-27063-4 34836955 PMC8626513

[74] Lavelle EC Ward RW. Mucosal vaccines — fortifying the frontiers. Nat Rev Immunol (2022) 22:236–50. doi: 10.1038/s41577-021-00583-2 PMC8312369 34312520

