# Supplementary material for: Post-vaccination T cell immunity to omicron
Source: Front Immunol. 2022 Jul 26;13:944713. doi: 10.3389/fimmu.2022.944713 (PMC9386871; doi:10.3389/fimmu.2022.944713)
Supplement: Supplementary file 1 [file DataSheet_1.pdf]

Supplementary table 1: Summary data on reviewed studies.

Author	Peer-rewiewed*	Primary regimen		Booster	Prior infection	Breakthrough infection	Hybrid immunity	number of samples	Age (median, range)	Sex distribution (% female)	fold-change CD4	fold-change CD8	Type of analysis	Comments
		Dose 1	Dose 2	Dose										
Keeton et al.	YES	Janssen - Ad26.CoV2.S	-	-	-	-	-	20	41 [not provided]	75	0.86	0.80	Frequency of spike-specific T cells producing any of the measured cytokines (IFN-γ, IL-2 or TNF) in response to peptide pools.	
		Janssen - Ad26.CoV2.S	-	Janssen - Ad26.CoV2.S	-	-	-	20	38 [not provided]	75	0.78	0.76		
		PfizerBioNTech - BNT162b2	PfizerBioNTech - BNT162b2	-	-	-	-	15	53 [not provided]	53	0.70	0.83		
		-	-	-	WT / Beta	-	-	15	39 [not provided]	12	0.77	0.75		
Tarke et al.	YES	mRNA	mRNA	-	-	-	-	19	not provided	not provided	0.84	0.85	$CD3^+CD4^+$ and $CD3^+CD8^+$ AIM staining with $OX40+CD137^+$ and $CD69^+CD137^+$ markers, respectively.	
De Marco et al	NO	multiple	multiple	-	-	-	-	10	43 [23-56]	70	0.84	0.83	Activation marker staining (CD69, CD40L for $CD4^+$ and CD69 and CD137 for $CD8^+$ T cells after peptide pool stimulation.	Data calculated from high-resolution figures; might not be 100% exact.
		mRNA	mRNA	mRNA	-	-	-	15	52 [26-60]	66	0.75	0.91		
		multiple	multiple	multiple (heterologous)	-	-	-	11	53 [26-62]	73	0.78	0.93		
		multiple	multiple	(multiple)	YES	-	YES	12	54 [22-60]	33	0.82	0.89		
		multiple	multiple	(multiple)	-	YES	YES	13	37 [21-54]	69	0.85	0.94		
Gao et al.	YES	PfizerBioNTech - BNT162b2	PfizerBioNTech - BNT162b2	-	-	-	-	40	53 [22-79]	58	0.91	0.92	$CD4^+$ T cell responses ($CD69^+CD154^+$) and $CD8^+$ T cell responses ($CD69^+CD137^+$) after peptide pool stimulation.	
		-	-	-	YES	-	-	26	54 [44-68]	31	0.84	0.70		
Liu et al.	YES	Janssen - Ad26.CoV2.S	-	-	-	-	-	20	43 [24-52]	55	0.97	0.86	IFN^+ $CD4^+$ and $CD8^+$ responses by ICS staining after peptide pool stimulation.	
		PfizerBioNTech - BNT162b2	PfizerBioNTech - BNT162b2	-	-	-	-	27	32 [22-67]	96	0.82	0.82		
Naranbhai et al.	YES	multiple	multiple	-	-	-	-	24	not provided	not provided	0.91	0.44	CD4+ and CD8+ CFSE (carboxyfluorescein succinimidyl ester (CFSE) proliferation assay) responses after peptide pool stimulation.	
		multiple	multiple	-	YES	-	YES	12			1.00	0.62		
		multiple	multiple	multiple (heterologous)	YES	-	YES	13			1.14	0.83		
Tan et al.	NO	PfizerBioNTech - BNT162b2	PfizerBioNTech - BNT162b2	-	-	-	-	41	36 [23-84]	78	1.00	0.94	IFN^+ $CD4^+$ and $CD8^+$ responses by ICS staining after peptide pool stimulation.	Data calculated with raw data (supplementary) including two-dose data from a booster graph. No raw data were provided for the booster analysis.
		PfizerBioNTech - BNT162b2	PfizerBioNTech - BNT162b2	Janssen - Ad26.CoV2.S	-	-	-	41			0.96	1.19		
		PfizerBioNTech - BNT162b2	PfizerBioNTech - BNT162b2	-	-	-	-	24	34 [23-69]	88	0.82	0.85		
		PfizerBioNTech - BNT162b2	PfizerBioNTech - BNT162b2	PfizerBioNTech - BNT162b2	-	-	-	24			0.89	1.18		
Geurtsvonkessel et al.	YES	AstraZeneca - Vaxzevria	AstraZeneca - Vaxzevria	-	-	-	-	15	63 [61-65]	60	0.58	0.18	$CD3^+CD4^+$ and $CD3^+CD8^+$ AIM staining with $OX40^+CD137^+$ and $CD69^+CD137^+$ markers, respectively.	Vaccine-specific data calculated from supplementary data only including subjects with reported measurements.
		Janssen - Ad26.CoV2.S	-	-	-	-	-	13	39 [20-58]	92	1.17	1.56		
		Moderna - mRNA-1273	Moderna - mRNA-1273	-	-	-	-	14	35 [26-57]	79	0.94	1.80		
		PfizerBioNTech - BNT162b2	PfizerBioNTech - BNT162b2	-	-	-	-	5	46 [36-57]	80	0.94	0.57		
		Moderna - mRNA-1273	Moderna - mRNA-1273	PfizerBioNTech - BNT162b2	-	-	-	9	29 [26-52]	78	5.21	19.70		
Schultz et al.	NO	Sinovac - CoronaVac	Sinovac - CoronaVac	Sinovac - CoronaVac	-	-	-	30	not provided	not provided	0.83	-	$CD3^+CD4^+$ AIM staining with $OX40^+CD137^+$.	
Madelon et al.	NO	mRNA	mRNA	-	-	-	-	20	46 [not provided]	45	0.62	0.79	$CD4^+$ and $CD8^+$ AIM staining with $OX40^+41\text{-}BB^+$ and $CD69^+41\text{-}BB^+$ markers, respectively.	anti-CD20-treated multiple sclerosis patients.
		mRNA	mRNA	mRNA	-	-	-	20			0.72	0.71		
Soto et al.	NO	Sinovac - CoronaVac	Sinovac - CoronaVac	-	-	-	-	32	not provided [3-17]	not provided	1.63	-	$CD3^+CD4^+$ AIM staining with $OX40^+CD137^+$.	3 - 17 year-old children.
Mazzoni et al.	YES	PfizerBioNTech - BNT162b2	PfizerBioNTech - BNT162b2	-	-	-	-	11	56 [23-65]	64	0.87	-	Spike-specific CD4+ T cells by ICS staining (IFN-γ and IL-2) after peptide pool stimulation.	
		PfizerBioNTech - BNT162b2	PfizerBioNTech - BNT162b2	PfizerBioNTech - BNT162b2	-	-	-	9	not provided	not provided	0.82	-		
Choi et al	YES	PfizerBioNTech - BNT162b2	PfizerBioNTech - BNT162b2	-	-	-	-	12	not provided	not provided	0.84	0.80	Spike-specific $CD4^+$ and $CD8^+$ T cells by ICS staining (IFN-γ) after peptide pool stimulation.	

* at submission of this review.
